# Improved quality of life of patients with generalized pustular psoriasis in Japan: A cross‐sectional survey

**DOI:** 10.1111/1346-8138.15657

**Published:** 2020-10-25

**Authors:** Koremasa Hayama, Hideki Fujita, Keiji Iwatsuki, Tadashi Terui

**Affiliations:** ^1^ Division of Cutaneous Science Department of Dermatology Nihon University School of Medicine Tokyo Japan; ^2^ Department of Dermatology Okayama University Graduate School of Medicine, Dentistry and Pharmaceutical Sciences Okayama Japan; ^3^ Department of Dermatology Fujita Health University School of Medicine Toyoake Japan

**Keywords:** biologics, epidemiology, generalized pustular psoriasis, quality of life, 36‐Item Short‐Form Health Survey version 2

## Abstract

Generalized pustular psoriasis (GPP) is a rare variant of psoriasis with severe clinical symptoms. However, the quality of life (QoL) of the patients is largely unknown. We conducted a nationwide cross‐sectional survey of Japanese GPP patients using the 36‐Item Short‐Form Health Survey version 2 (SF‐36v2) to elucidate patients’ QoL and how their QoL had changed over the last decade. We analyzed QoL data of 83 patients obtained from 2016 to 2019 (present group) and compared it with that of 105 patients collected in a previous survey conducted between 2003 and 2007 (past group). Although the QoL of the present patients was still largely impaired in comparison with the standard Japanese population, substantial improvement was found in some SF‐36v2 subscales including “general health”, “vitality”, “social functioning” and “mental health” as compared with that of the past group. Advances in treatment may contribute to this QoL improvement.

## Introduction

Generalized pustular psoriasis (GPP) is a rare variant of psoriasis, which represent 2% of psoriatic diseases.^1^ It is characterized by recurrent fever and systemic flushing accompanied by extensive sterile pustules.^1^ Clinical symptoms related to systemic inflammation may also develop during the course of the disease. In addition, GPP is often complicated by mucosal manifestations and arthritis, and occasionally by respiratory failure, ocular symptoms and secondary amyloidosis.^2^ Therefore, GPP has a negative impact on patients’ quality of life (QoL).

Because of the small number of GPP patients in each facility, little is known about their QoL. Although no studies investigating the QoL of GPP patients were retrieved from the PubMed database, a cross‐sectional QoL survey on Japanese patients with GPP using the Medical Outcomes Study 36‐Item Short‐Form Health Survey version 2 (SF‐36v2) was carried out by a group from Okayama University between 2003 and 2007.^3^ According to the survey, most patients showed relatively low scores in “general health”, “social functioning” and “role‐emotional” among the subscales of SF‐36v2, indicative of impaired QoL of GPP patients.^3^ On the other hand, it can be estimated that patients’ QoL has improved through the development of new treatments and the establishment of guidelines over the past years.

The purpose of this study was to elucidate the QoL of present GPP patients by conducting a cross‐sectional survey using SF‐36v2 and to examine how the QoL of the patients has changed over the last decade by comparing past (2003–2007) and present (2016–2019) data.

## Methods

A questionnaire‐based study was performed by sending questionnaires to the 668 hospitals/facilities providing dermatological training under the certification of the Japanese Dermatological Association. The propriety of the participation in this research and the patient number were verified by the first questionnaire. The second questionnaire was sent to the participating institutions to collect the data of patients who were diagnosed with GPP based on the diagnostic criteria of Japanese guidelines.^2^ Data on present patients’ age, sex, clinical symptoms, laboratory findings and QoL assessed by the Japanese‐language version of SF‐36v2^4^, ^5^, ^6^ were obtained from 2016 to 2019. SF‐36v2 is currently the most internationally used comprehensive health‐related QoL scale^7^ that is applicable to various types of disease. SF‐36v2 includes 36 questions, in a Likert‐type or forced‐choice format, intended to measure the following eight dimensions of health: “physical functioning”, “role‐physical”, “bodily pain”, “general health”, “vitality”, “social functioning”, “role‐emotional” and “mental health”.^8^ The past QoL data were collected through the survey conducted by the group from Okayama University using SF‐36v2 between 2003 and 2007, in which 47 facilities providing dermatological training under the certification of the Japanese Dermatological Association were enrolled. The analyzed data have been publicly available on the Ministry of Health, Labor and Welfare’s website.^3^ To compare patients’ data with those of coeval general Japanese people, we used the national standard values of Japan in 2007 and 2017 provided by i‐Hope International (Kyoto, Japan). The value of each SF‐36v2 element from the national standard population was adjusted to the average of 50 points with 10 points standard deviation (SD).^7^ Then, the values of individual SF‐36v2 elements of the GPP patients were converted accordingly to obtain T‐scores.

This study was approved by the ethics committee of Nihon University Itabashi Hospital (RK‐151110‐3). The SF‐36v2 license for this study was obtained from i‐Hope International.

Statistical analyses were performed using GraphPad Prism version 8 (GraphPad Software, La Jolla, CA, USA), in which *P* < 0.05 is considered statistically significant. The *Z*‐test was performed for comparison with the standard population. Differences between the two groups were analyzed using the Mann–Whitney *U*‐test. Fisher’s exact test was employed to examine differences in patient backgrounds.

## Results

A total of 83 patients’ data (45 males and 38 females) from 33 institutions were collected for analyses as the present group. The past group consisted of 105 patients (36 males and 69 females). Most of the patients were classified as von Zumbusch type, 99 (94.3%) in the past group and 80 (96.4%) in the present group based on the Japanese guidelines.^2^ Detailed patient backgrounds are shown in Table 1.

**Table 1:** Backgrounds of the past and present patients

	Past (2003–2007)	Present (2016–2019)	*P*
No. of patients			
Total	105	83	
Male	36 (34.3%)	45 (54.2%)	0.0076*
Female	69 (65.7%)	38 (45.8%)	
Mean age (years ± SD)			
Total	53.42 ± 17.71	55.84 ± 20.94	0.2964
Male	54.27 ± 16.27	56.73 ± 20.48	0.3376
Female	52.96 ± 18.42	54.79 ± 21.44	0.6388
Subgroup			
Von Zumbusch type	99 (94.3%)	80 (96.4%)	0.479
Impetigo herpetiformis	2 (1.9%)	0	
Acrodermatitis continua of Hallopeau	1 (1.0%)	0	
Unknown	3 (2.9%)	3 (3.6%)	
Treatment			
Topical treatment			
Steroid	82 (78.1%)	52 (63.8%)	
Active vitamin D_3_	60 (57.1%)	29 (36.1%)	
Oral medication			
Etretinate	53 (50.8%)	24 (28.9%)	
Cyclosporin	38 (36.2%)	16 (19.3%)	
Methotrexate	12 (11.4%)	12 (14.6%)	
Oral steroid	18 (17.1%)	13 (15.7%)	
Biologics			
Infliximab	0	22 (26.5%)	
Adalimumab	0	3 (3.6%)	
Ustekinumab	0	2 (2.4%)	
Secukinumab	0	12 (14.5%)	
Brodalumab	0	1 (1.2%)	
Ixekizumab	0	1 (1.2%)	
Others			
Ultraviolet treatment	9 (8.5%)	1 (1.2%)	
Granulocyte and monocyte adsorption apheresis	0	6 (7.2%)	

*P < 0.05. SD, standard deviation.

Treatment backgrounds of the patients are also summarized in Table 1. In the present group, 41 (51.3%) patients were on biologic treatment, in contrast to none in the past population. Similarly, granulocyte and monocyte adsorption apheresis (GMA) was performed only in six (7.2%) present patients. At the time of the previous survey (2003–2007), biologics and GMA were not approved for psoriatic diseases in Japan. Thus, treatment backgrounds were considerably different between past and present arms.

**Figure 1:**
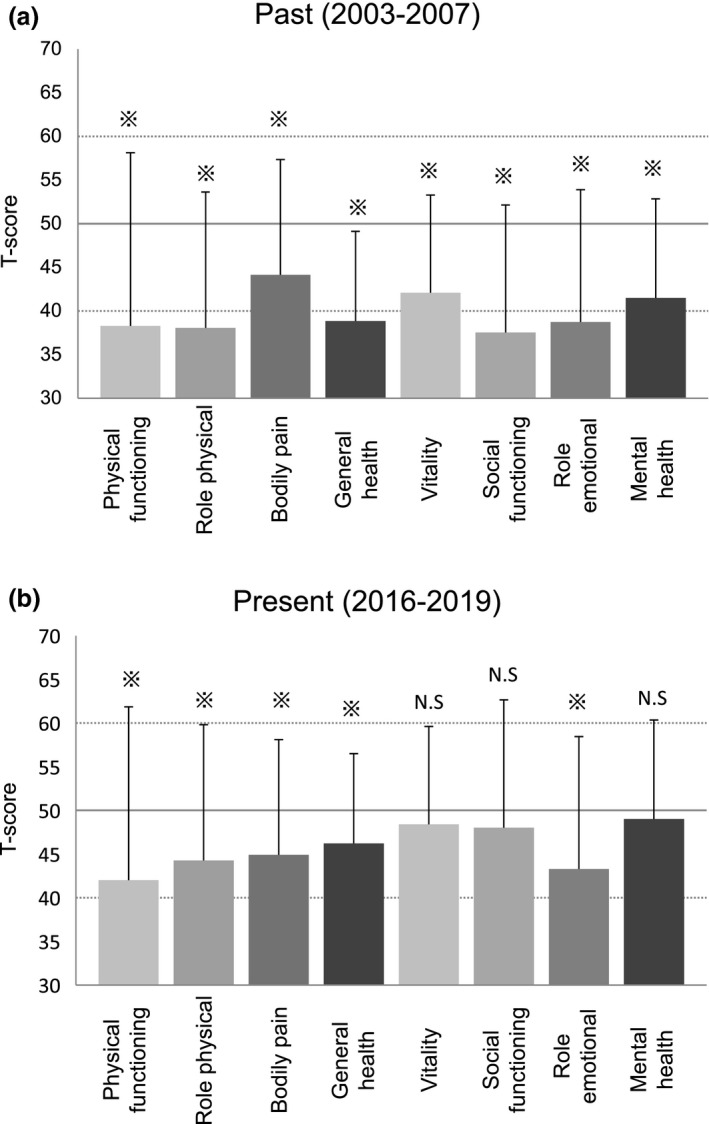
Impaired quality of life of past and present generalized pustular psoriasis patients. (a) Quality of life values of the past (2003–2007) patients. T‐scores for each 36‐Item Short‐Form Health Survey version 2 (SF‐36v2) element were calculated from 2007 Japanese national standard values. (b) Quality of life values of the present (2016–2019) patients. T‐scores for each SF‐36v2 element were calculated from 2017 Japanese national standard values. (※) *P *< 0.05 compared with national standard value. N.S, not significant.

Each SF‐36v2 element from the national standard population in 2007 and 2017 was adjusted to the average of 50 points with 10 points SD. Then, patients’ data of the individual elements from past and present populations were converted accordingly into T‐scores for comparison. All SF‐36v2 subscales of the past group showed significantly lower values than the 2007 national standard (Fig. 1a). Whereas present patients also exhibited significantly lower scores for five out of eight subscales, their statuses of vitality, social functioning and mental health were comparable with those of the 2017 standard population (Fig. 1b).

We next examined how patients’ QoL changed over the last decade and calculated T‐scores on the basis of the 2007 national standard for both past and present arms. It was found that the T‐scores of the present group were statistically significantly higher in general health, vitality, social functioning and mental health (Table 2). For the sake of reference, we also compared T‐scores of the past patients calculated based on the 2007 national standard and those of the present patients determined from the 2017 national standard (Table S1). Interestingly, all SF‐36v2 subscales except bodily pain were statistically significantly improved from the past group. Taken together, although still impaired, substantial improvement of QoL in GPP patients was demonstrated.

**Table 2:** Comparison of T‐scores of SF‐36v2 subscales calculated from 2007 Japanese standard between past and present patients

	Past (2003–2007)	Present (2016–2019)	*P*
Physical functioning	38.28 ± 19.82	42.59 ± 20.02	0.1741
Role‐physical	38.05 ± 15.55	41.89 ± 14.69	0.0705
Bodily pain	44.13 ± 13.20	45.80 ± 12.54	0.481
General health	38.84 ± 10.27	43.53 ± 8.97	0.0004*
Vitality	42.07 ± 11.20	45.09 ± 10.67	0.0417*
Social functioning	37.51 ± 14.63	46.66 ± 12.54	<0.0001*
Role‐emotional	38.74 ± 15.13	40.94 ± 15.38	0.1559
Mental health	41.50 ± 11.34	45.83 ± 12.46	0.0089*

*P < 0.05. SF‐36v2, 36‐Item Short‐Form Health Survey version 2.

## Discussion

This is the first QoL study exclusively focused on GPP patients. In the data of the past group, all T‐scores of the subscales were significantly lower than the average of standard Japanese people. During the last decade, half of the subscales (general health, vitality, social functioning and mental health) were statistically significantly improved. Intact statuses of vitality, social functioning and mental health subscales only in the present patients may also support QoL improvement. In reference, comparison using standard populations in 2007 for past and 2017 for present patients, all subscales but bodily pain were statistically significantly improved. Although this is just a reference calculation approach, it suggests the possibility that wider ranges of QoL elements may have improved.

One possible explanation for this QoL improvement is recent advances in treatment. New treatments such as biologics and GMA were first approved for GPP in 2010 and 2012, respectively, in Japan. Indeed, some treatments were reported to improve QoL of GPP patients.^9^, ^10^ However, the scores of five out of the eight subscales were still lower than standard values in the present patients. In particular, T‐scores for role‐physical and role‐emotional remained very low, indicating that work and active daily life were still considerably hindered. Further treatment advances may be required for the better QoL of GPP patients.

Our study has some limitations. First, there were some differences in patient backgrounds between the past and present populations. There was a substantial difference in sex ratio between the past and present groups for an unknown reason. Second, we could not compare the disease severity data between past and present arms, because Japanese GPP guidelines revised the severity criteria in 2014.^2^ Third, QoL data of the general population change over time. Thus, it is not easy to make a simple comparison of QoL data obtained at different periods. Fourth, the effectiveness of each treatment was not taken into consideration. Although no statistically significant differences were found between biologic users and non‐users in all QoL subscales in the present patients (data not shown), prospective studies are needed to examine how initial disease severity and each treatment affect patients’ QoL during the long course of the disease.

In summary, this is the first study showing that GPP patients have impaired QoL compared with controls, even with recent advances in the treatment. We also demonstrated for the first time that QoL of GPP patients significantly improved during the last decade. Further researches are required to elucidate the contribution of new treatments such as biologics to patients’ QoL.

## Conflict of Interest

K. H. reports grants, personal fees and non‐financial support from AbbVie; grants and personal fees from Eisai; personal fees from Janssen Pharmaceutical; grants and personal fees from Kaken Pharmaceutical; grants and personal fees from Kyorin; grants and personal fees from Maruho; grants and personal fees from Mitubishi‐Tanabe; grants from Nihon Pharmaceutical; grants, personal fees and non‐financial support from Novartis; grants and personal fees from Sanofi; grants from Sun Pharma; grants and personal fees from Taiho; and grants from Kyowa‐Kirin, outside the submitted work. H. F. reports grants and personal fees from AbbVie; grants and personal fees from Eisai; personal fees from Janssen Pharmaceutical; grants from Kaken Pharmaceutical; grants from Kyorin; grants and personal fees from Maruho; grants and personal fees from Mitubishi‐Tanabe; grants from Nihon Pharmaceutical; grants and personal fees from Novartis; grants and personal fees from Sanofi; grants from Sun Pharma; grants and personal fees from Taiho; personal fees from Eli Lilly; grants and personal fees from Kyowa‐Kirin; personal fees from UCB; and personal fees from Boehringer Ingelheim, outside the submitted work. K. I. reports personal fees from Minophagen; personal fees from NAOS JAPAN; personal fees from GSK; personal fees from Kyowa‐Kirin; personal fees from Torii Pharmaceutical; and personal fees from Mitsubishi‐Tanabe, outside the submitted work. T. T. reports grants and personal fees from AbbVie; grants and personal fees from Eisai; personal fees from Janssen Pharmaceutical; grants from Kaken Pharmaceutical and Kyorin; grants and personal fees from Maruho and Mitubishi‐Tanabe; grants from Nihon Pharmaceutical; grants and personal fees from Novartis; grants from Sanofi; grants from Sun Pharma; grants and personal fees from Taiho; personal fees from Eli Lilly; grants and personal fees from Kyowa‐Kirin; and personal fees from Boehringer Ingelheim, outside the submitted work.

## Supporting information


**Table S1.** Comparison of T‐scores of 36‐Item Short‐Form Health Survey version 2 (SF‐36v2) subscales calculated from respective coeval Japanese standards between past and present patients
