# Supplementary material for: Improved quality of life of patients with generalized pustular psoriasis in Japan: A cross‐sectional survey
Source: J Dermatol. 2020 Oct 25;48(2):203–6. doi: 10.1111/1346-8138.15657 (PMC7894492; doi:10.1111/1346-8138.15657)
Supplement: Supplementary file 1 — Table S1. Comparison of T‐scores of 36‐Item Short‐Form Health Survey version 2 (SF‐36v2) subscales calculated from respective coeval Japanese standards between past and present patients [file JDE-48-203-s001.pptx]

## Slide 1
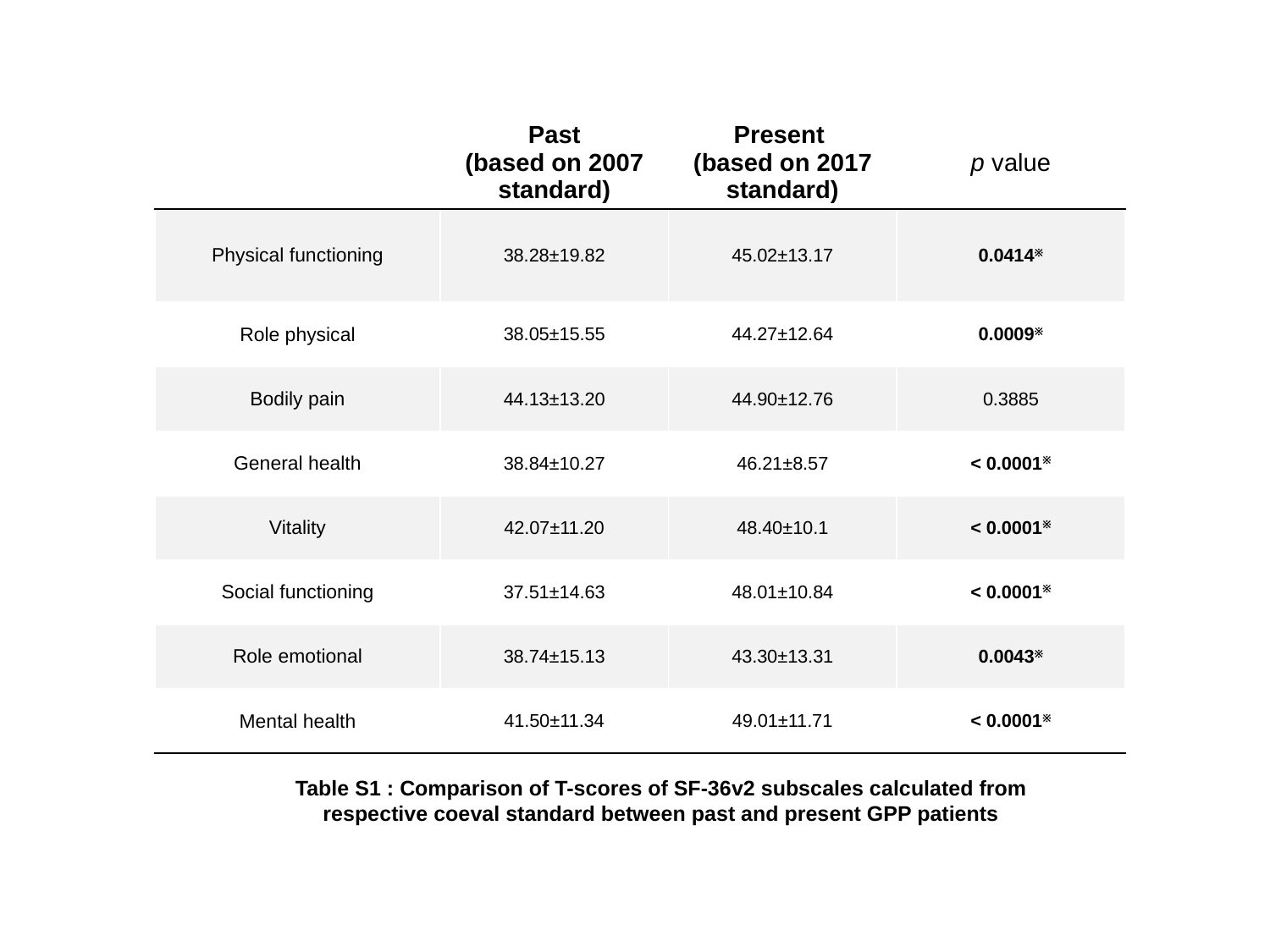

| | Past (based on 2007 standard) | Present (based on 2017 standard) | p value |
| --- | --- | --- | --- |
| Physical functioning | 38.28±19.82 | 45.02±13.17 | 0.0414※ |
| Role physical | 38.05±15.55 | 44.27±12.64 | 0.0009※ |
| Bodily pain | 44.13±13.20 | 44.90±12.76 | 0.3885 |
| General health | 38.84±10.27 | 46.21±8.57 | < 0.0001※ |
| Vitality | 42.07±11.20 | 48.40±10.1 | < 0.0001※ |
| Social functioning | 37.51±14.63 | 48.01±10.84 | < 0.0001※ |
| Role emotional | 38.74±15.13 | 43.30±13.31 | 0.0043※ |
| Mental health | 41.50±11.34 | 49.01±11.71 | < 0.0001※ |
Table S1 : Comparison of T-scores of SF-36v2 subscales calculated from respective coeval standard between past and present GPP patients
